# Modeling the Spread of COVID-19 with the Control of Mixed Vaccine Types during the Pandemic in Thailand

**DOI:** 10.3390/tropicalmed8030175

**Published:** 2023-03-16

**Authors:** Tanatorn Intarapanya, Apichat Suratanee, Sittiporn Pattaradilokrat, Kitiporn Plaimas

**Affiliations:** 1 Advanced Virtual and Intelligence Computing (AVIC) Center, Department of Mathematics and Computer Science, Faculty of Science, Chulalongkorn University, Bangkok 10330, Thailand; tanatorn.int@hotmail.com; 2 Department of Mathematics, Faculty of Applied Science, King Mongkut’s University of Technology North Bangkok, Bangkok 10800, Thailand; apichat.s@sci.kmutnb.ac.th; 3 Intelligent and Nonlinear Dynamic Innovations Research Center, Science and Technology Research Institute, King Mongkut’s University of Technology North Bangkok, Bangkok 10800, Thailand; 4 Department of Biology, Faculty of Science, Chulalongkorn University, Bangkok 10330, Thailand; sittiporn.p@chula.ac.th

**Keywords:** mathematical model, COVID-19, vaccine

## Abstract

COVID-19 is a respiratory disease that can spread rapidly. Controlling the spread through vaccination is one of the measures for activating immunization that helps to reduce the number of infected people. Different types of vaccines are effective in preventing and alleviating the symptoms of the disease in different ways. In this study, a mathematical model, *SVIHR*, was developed to assess the behavior of disease transmission in Thailand by considering the vaccine efficacy of different vaccine types and the vaccination rate. The equilibrium points were investigated and the basic reproduction number $\left(R_{0}\right)$ was calculated using a next-generation matrix to determine the stability of the equilibrium. We found that the disease-free equilibrium point was asymptotically stable if, and only if, $R_{0}<1$, and the endemic equilibrium was asymptotically stable if, and only if, $R_{0}>1$. The simulation results and the estimation of the parameters applied to the actual data in Thailand are reported. The sensitivity of parameters related to the basic reproduction number was compared with estimates of the effectiveness of pandemic controls. The simulations of different vaccine efficacies for different vaccine types were compared and the average mixing of vaccine types was reported to assess the vaccination policies. Finally, the trade-off between the vaccine efficacy and the vaccination rate was investigated, resulting in the essentiality of vaccine efficacy to restrict the spread of COVID-19.

## 1. Introduction

Severe acute respiratory syndrome coronavirus 2 (SARS-CoV-2) is a viral pathogen that was first reported in December 2019 in Wuhan, China [1]. The World Health Organization (WHO) named the disease caused by this virus coronavirus disease 2019 (COVID-19) [2]. The report from the World Health Organization in February 2023 revealed 756 million cases of COVID-19 worldwide and 6.8 million deaths [3], negatively affecting public health and economic systems all around the world. The symptoms of this disease in non-immune individuals are generally high fever, cough, headache, muscle aches, sore throat, difficulty breathing, and fatigue. In some cases, patients lose their sense of taste or smell. When treatments are not available, a small number of patients can develop severe complications, including severe shortness of breath, persistent pain in the chest, and even death [4,5]. The virus can spread quickly through mouth or nose droplets from coughing or sneezing. The virus can survive on hard surfaces, such as plastic and stainless steel, for up to 72 h [6], causing widespread outbreaks in many countries. Thus, current measures to control the spread include social distancing and stimulating immunization of the population through vaccination [7].

Vaccines are used to generate immunity in the body. Seven vaccines against COVID-19 have been approved for vaccination in Thailand, categorized into four types according to the vaccine technologies [8]. The first type is an inactivated vaccine, such as the Sinovac–CoronaVac COVID-19 vaccine [9] and the Sinopharm vaccine [10]. This type of vaccine is developed by growing the virus in vitro, then the virus is killed to remove the disease’s capacity while retaining the ability to stimulate immune responses. The second type is a viral vector vaccine that is made from other non-replicating viruses, serving as a carrier for the expression of the genetic material of the SARS-CoV-2 spike protein. Two types of the adenovirus vector vaccine are available in Thailand, including the ChAdOx1 vaccine (Oxford–AstraZeneca) [10] and the Janssen COVID-19 vaccine [11]. The third type is an mRNA vaccine obtained by synthesizing the genetic code mRNA that encodes the viral spike protein, such as BNT162b2 (Pfizer–BioNTech) and mRNA-1273 vaccine (Moderna) [12]. The delivery of the vaccine is achieved by encapsulating the mRNA inside nanoparticles, which help stabilize the vaccine components and promote absorption into cells. The fourth type is a protein subunit vaccine, such as the Novavax vaccine, developed by expressing the spike protein in insect cells [13]. The vaccines against COVID-19 are licensed by the World Health Organization for prophylaxis in many countries; to date, over 12 billion doses have been administered, and 5 billion people (60% of the global population) have been injected with one dose of vaccine worldwide [3]. In Thailand, 57 million people (80% of the Thai population) have been injected with one of the COVID-19 vaccines [14]. The vaccine components are made differently, which could elicit immunity through different cellular pathways [15]; therefore, the efficacies in preventing severe symptoms of COVID and preventing death from the disease are different.

Several types of mathematical models are used to study the behavior of epidemics. The simplest and most popular fundamental model is the *SIR* model [16,17,18], which describes the change in population in the system through differential equations by dividing the population into three classes: susceptible class $\left(S\right)$, infected class $\left(I\right)$, and recovered class $\left(R\right)$. A more complex model is also available where populations are grouped into multiple statuses with increased disease control factors, such as vaccination control [19] and convalescent plasma transfusion [20]. In 2020, Din et al. [21] developed a mathematical model for COVID-19 in Thailand which divided the population into six classes, namely, the *SLIQHR* model. They studied the latent population, which is an asymptomatic group, but vaccination control was not considered. In 2021, Husniah et al. [20] demonstrated the SEIR model of convalescent plasma transfusion as a control to reduce the spread of COVID-19. The effect of convalescent plasma transfusion helps to increase patient survival [20]. In 2022, Intarapanya et al. considered the spread of COVID-19 in Thailand due to immigration, using a model referred to as *LUSIHR* [22]. This model consists of six classes including legal immigrants, undocumented immigrants, susceptible people, infected people, hospitalized people, and recovered people. To control the spread of the disease, the use of vaccines is the primary choice to establish herd immunity and reduce the numbers of cases and deaths. Convalescent plasma from infected people is another choice to protect and treat people from the disease [23]. Although convalescent plasma transfusion is an effective way to treat people infected with COVID-19, it has many limitations, such as a lack of neutralizing antibodies in patient plasma, large infusion volumes, time of administration of the plasma to infected patients, etc. [24]. As COVID-19 vaccines are being deployed worldwide, the qualitative development and analysis of a COVID-19 mathematical model, considering available vaccination data for susceptible people, has been studied continually. In 2020, Diagne et al. [25] developed a mathematical model of COVID-19 with vaccination control where vaccinated people could be infected according to vaccine efficacy. In 2021, Giulia et al. [19] developed a vaccination model for the COVID-19 pandemic in Italy, namely, SIDARTHE-V. This model considered both symptomatic and asymptomatic people combined with their diagnoses. Mathematical models related to the spread of pathogens among populations have continued to evolve and are very useful in research. In particular, consideration should be given to vaccination measures aiming to prevent and control the spread of disease.

Determining the efficacy of vaccines using mathematical models for the spread of COVID-19 in Thailand is still not straightforward with the limitation of collected data and the need for parameters in modeling to be as consistent with reality as possible. This paper proposes a mathematical model that considers the vaccinated population. The model was simplified to be consistent with the population and available data in Thailand. The probability of re-infection of recovered individuals is omitted due to a lack of information and to simplify the model. We studied the positivity of the solution, equilibrium points, the basic reproduction number, and the stability of the equilibrium. Moreover, simulations of the equilibrium points were undertaken. We also estimated the values of parameters fitted with the actual data and studied the trade-off between the vaccination rate and vaccine efficacy.

## 2. Materials and Methods

### 2.1. Mathematical Models

Our model is based on the *SIR* model [16,17,18] and the vaccination model of Diagne et al. [25] which assume that vaccinated individuals can be infected depending on the vaccine efficacy. The model is named the susceptible-vaccinated-infected-hospitalized-recovered (*SVIHR*) model. We considered three types of vaccination in Thailand: RNA-based (mRNA), non-replicating viral vector, and inactivated virus to compare the efficiency for each type.

**Figure 1:**
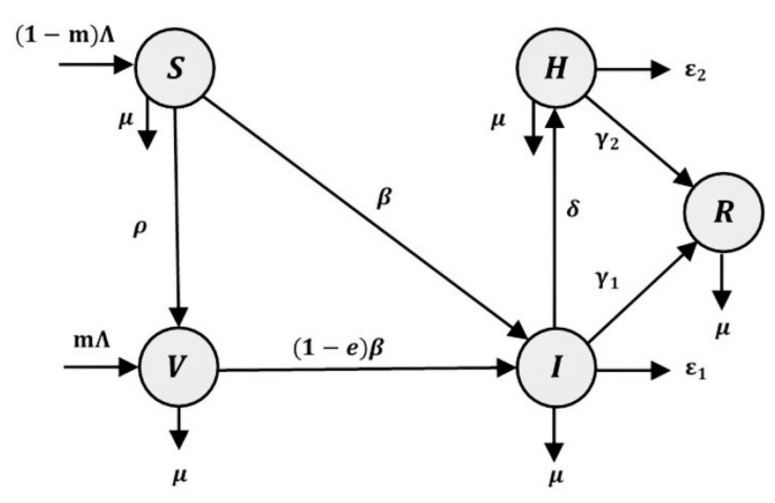
A flowchart of the susceptible-vaccinated-infected-hospitalized-recovered (*SVIHR*) model.

The *SVIHR* model divides the population into five subpopulations: susceptible $\left(S\right)$ although not infected; vaccinated individuals $\left(V\right)$; infected individuals $\left(I\right)$ who are infected but do not have to stay in hospital; hospitalized individuals $\left(H\right)$ who tested positive (detected) and stay in hospital; and recovered individuals $\left(R\right)$, who recover from the disease and are unable to be infected again. A diagram of the *SVIHR* model is shown in Figure 1.

The relationships among subgroups of the population represent the flow of people in each subgroup. Susceptible people are those in the population who are at risk of becoming infected with COVID-19. Therefore, a fraction of the susceptible group can become infected individuals and the rest could be vaccinated to protect against the disease. However, some vaccinated people are still susceptible to infection, but with a lower probability. Therefore, a proportion of vaccinated people could become infected people. In the flowchart of the population in the system, infected people represent the central component in the diagram. The number of infected people could be increased when susceptible or vaccinated people are infected and could be reduced when the infected people have moved into a hospital to cure the disease, or into a recovered group after the disease has disappeared. The flow of the *SVIHR* model is described by a system of differential equations, as follows:

(1) $$S^{\prime }\left(t\right)=\left(1-m\right)\Lambda -\beta S\left(t\right)I\left(t\right)-\rho S\left(t\right)-\mu S\left(t\right)$$

(2) $$V^{\prime }\left(t\right)=m\Lambda +\rho S\left(t\right)-\left(1-e\right)\beta V\left(t\right)I\left(t\right)-\mu V\left(t\right)$$

(3) $$I^{\prime }\left(t\right)=\beta S\left(t\right)I\left(t\right)+\left(1-e\right)\beta V\left(t\right)I\left(t\right)-\delta I\left(t\right)-\gamma_{1}I\left(t\right)-\varepsilon_{1}I\left(t\right)-\mu I\left(t\right)$$

(4) $$H^{\prime }\left(t\right)=\delta I\left(t\right)-\gamma_{2}H\left(t\right)-\varepsilon_{2}H\left(t\right)-\mu H\left(t\right)$$

(5) $$R^{\prime }\left(t\right)=\gamma_{1}I\left(t\right)+\gamma_{2}H\left(t\right)-\mu R\left(t\right)$$

where all parameters are positive numbers, and

Λ is the number of people recruited from the population per day;
m is the proportion of vaccinated individuals;
ρ is the vaccination rate per day;
e is the vaccine efficacy or reduced rate of vaccination, the value of which varies depending on the type of vaccine;
β is the transmission rate, the value of which is defined by the probability of disease transmission in a single contact multiplied by the average number of contacts per person;
$\gamma_{1},\;\gamma_{2}$ are the recovery rates of infected individuals and hospitalized individuals, respectively;
$\varepsilon_{1},\varepsilon_{2}$ are the induced COVID-19 death rates of infected individuals and hospitalized individuals, respectively;
δ is the rate of detection, which is the level of attention to the disease; and
μ is the natural death rate, i.e., the rate of people who die without COVID-19 symptoms.

### 2.2. Model Analysis

#### 2.2.1. The Nature of the Model Parameters

First, it is necessary to prove that the population numbers should not be negative. The idea of this proof is that the population will increase or stay the same at zero. The results are shown below:

Equation (1) is for susceptible people, $S\left(t\right)$, and $S^{\prime }\left(t\right)=\left(1-m\right)\Lambda -\beta S\left(t\right)I\left(t\right)-\rho S\left(t\right)-\mu S\left(t\right)$. Let the initial population be non-negative, $S\left(0\right)\geq 0$. For some $t_{s}\geq 0$, $S\left(t_{s}\right)=0$, then $S^{\prime }\left(t_{s}\right)=\left(1-m\right)\Lambda >0$ since m is the proportion of vaccinated individuals and Λ is the number of recruited individuals. This means that, for every point *t_s_* when *S*(*t*_s_) = 0, the rate of change for *S*(*t*_s_) is positive ($S^{\prime }\left(t_{s}\right)>0$) which results in increasing values of *S*(*t*) when *t* > *t_s_*. Therefore, $\underset{t\to t_{s}^{+}}{\lim}S\left(t\right)>0$. Hence, $S\left(t\right)\geq 0$ for all t≥0.

Equation (2) is for the vaccinated population $V\left(t\right)$ and $V^{\prime }\left(t\right)=m\Lambda +\rho S\left(t\right)-\left(1-e\right)\beta V\left(t\right)I\left(t\right)-\mu V\left(t\right)$. Let the initial population be non-negative, $V\left(0\right)\geq 0$. For some $t_{v}\geq 0$, $V\left(t_{v}\right)=0$, then $V^{\prime }\left(t_{v}\right)=m\Lambda +\rho S\left(t_{v}\right)$. ρ is the vaccination rate and $S\left(t\right)\geq 0$ for all t≥0. Therefore, $\underset{t\to t_{v}^{+}}{\lim}V\left(t\right)>0$. Hence, $V\left(t\right)\geq 0$ for all t≥0.

Equation (3) is for the infected population, $I\left(t\right)$, and $I^{\prime }\left(t\right)=\beta S\left(t\right)I\left(t\right)+\left(1-e\right)\beta V\left(t\right)I\left(t\right)-\delta I\left(t\right)-\gamma_{1}I\left(t\right)-\varepsilon_{1}I\left(t\right)-\mu I\left(t\right)$. Let the initial population be non-negative, $I\left(0\right)\geq 0$. For some $t_{i}\geq 0$, $I\left(t_{i}\right)=0$, then $I^{\prime }\left(t_{i}\right)=0$. Therefore, $\underset{t\to t_{i}^{+}}{\lim}I\left(t\right)=0$. Hence, $I\left(t\right)\geq 0$ for all t≥0.

Equation (4) is for the hospitalized population, $H\left(t\right)$, and $H^{\prime }\left(t\right)=\delta I\left(t\right)-\gamma_{2}H\left(t\right)-\varepsilon_{2}H\left(t\right)-\mu H\left(t\right)$. Let the initial population be non-negative, $H\left(0\right)\geq 0$. For some $t_{h}\geq 0$, $H\left(t_{h}\right)=0$, then $H^{\prime }\left(t_{h}\right)=\delta I\left(t_{h}\right)\geq 0$ since $I\left(t\right)\geq 0$ for all t. Therefore, $\underset{t\to t_{h}^{+}}{\lim}H\left(t\right)\geq 0$. Hence, $H\left(t\right)\geq 0$ for all t≥0.

In conclusion, all populations are non-negative for all t≥0; thus, this model can be applied to analyze the equilibrium points and the stability.

#### 2.2.2. Equilibrium Points

We set all differential equations (Equations (1)–(5)) to zero in the following system:

(6) $$S^{\prime }\left(t\right)=\left(1-m\right)\Lambda -\beta S\left(t\right)I\left(t\right)-\rho S\left(t\right)-\mu S\left(t\right)=0$$

(7) $$V^{\prime }\left(t\right)=m\Lambda +\rho S\left(t\right)-\left(1-e\right)\beta V\left(t\right)I\left(t\right)-\mu V\left(t\right)=0$$

(8) $$I^{\prime }\left(t\right)=\beta S\left(t\right)I\left(t\right)+\left(1-e\right)\beta V\left(t\right)I\left(t\right)-\delta I\left(t\right)-\gamma_{1}I\left(t\right)-\varepsilon_{1}I\left(t\right)-\mu I\left(t\right)=0$$

(9) $$H^{\prime }\left(t\right)=\delta I\left(t\right)-\gamma_{2}H\left(t\right)-\varepsilon_{2}H\left(t\right)-\mu H\left(t\right)=0$$

(10) $$R^{\prime }\left(t\right)=\gamma_{1}I\left(t\right)+\gamma_{2}H\left(t\right)-\mu R\left(t\right)=0$$

The solutions of the system of Equations (6)–(10) are the equilibrium points. We obtained two equilibrium points: (1) the disease-free equilibrium point (DFE), where the infection disappears from the system; and (2) the endemic equilibrium point (EE), where the infection still occurs in the system. These equilibrium points are shown as follows.

The DFE is

(11) $$E_{dfe}=\left(\;S_{1}^{*},\;V_{1}^{*},\;0,\;0,\;0\right),$$

where $S_{1}^{*}=\frac{\left(1-m\right)\Lambda }{B}$, $V_{1}^{*}=\frac{m\Lambda +\rho S_{1}^{*}}{\mu }$.

The EE point is

(12) $$E_{end}=\left(\;S_{2}^{*},V_{2}^{*},\;I_{2}^{*},\;H_{2}^{*},\;R_{2}^{*}\right),$$

where $S_{2}^{*}=\frac{\left(1-m\right)\Lambda }{\beta I_{2}^{*}+B}$, $V_{2}^{*}=\frac{A}{\left(1-e\right)\beta }-\frac{S_{2}^{*}}{1-e}$, $I_{2}^{*}=\frac{-b+\sqrt{b^{2}-4ac}}{2a}$, $H_{2}^{*}=\frac{\delta I_{2}^{*}}{C}$, $R_{2}^{*}=\frac{\gamma_{1}I_{2}^{*}+\gamma_{2}H_{2}^{*}}{\mu }$,

with $A=\delta +\varepsilon_{1}+\gamma_{1}+\mu$, B=ρ+μ, $C=\gamma_{2}+\varepsilon_{2}+\mu$, $a=\left(1-e\right)A\beta$, $b=A\mu +\left(1-e\right)AB-\left(1-e\right)\Lambda \beta$, $c=\frac{AB\mu }{\beta }-\left(1-m\right)\Lambda \mu -\left(1-e\right)\Lambda \rho -\left(1-e\right)\Lambda m\mu$, $b^{2}-4ac\geq 0$ and $-b+\sqrt{b^{2}-4ac}>0$.

#### 2.2.3. The Basic Reproduction Numbers

The basic reproduction number $\left(R_{0}\right)$ is the average number of cases generated per infected individual [26]. When $R_{0}>1$, the infection will spread in a trajectory that converges to the EE state. However, when $R_{0}<1$, the infection will decrease over time and the number of infected people will reach the DFE state. We used the next-generation matrix [27] to determine $R_{0}$. First, we define

(13) $$F=\left[\begin{array}{c} \beta SI+\left(1-e\right)\beta VI\\ 0 \end{array}\right]\;\mathrm{and}\;\chi =\left[\begin{array}{c} AI\\ -\delta I+CH \end{array}\right]$$

The Jacobian matrices of F and χ are denoted as F and X, respectively.

(14) $$F=\left[\begin{array}{cc} \beta S_{1}^{*}+\left(1-e\right)\beta V_{1}^{*} & 0\\ 0 & 0 \end{array}\right]$$

(15) $$X=\left[\begin{array}{cc} A & 0\\ -\delta & C \end{array}\right],\;X^{-1}=\frac{1}{AC}\left[\begin{array}{cc} C & 0\\ \delta & A \end{array}\right]$$

The spectral radius of the next-generation matrix $FX^{-1}$ is the basic reproduction number $R_{0}$.

(16) $$FX^{-1}=\left[\begin{array}{cc} \frac{\beta S_{1}^{*}+\left(1-e\right)\beta V_{1}^{*}}{A} & 0\\ 0 & 0 \end{array}\right]$$

The eigenvalues of $FX^{-1}$ are $\lambda_{1}=0$ and $\lambda_{2}=\frac{\beta S_{1}^{*}+\left(1-e\right)\beta V_{1}^{*}}{A}$.

(17) $$R_{0}=\rho \left(FX^{-1}\right)=\max\left\{\left|\lambda_{1}\right|,\left|\lambda_{2}\right|\right\}=\frac{\beta \Lambda \left[\mu +\rho -me\mu -e\rho \right]}{\left(\delta +\varepsilon_{1}+\gamma_{1}+\mu \right)\left(\rho +\mu \right)\mu }.$$

Equation (17) represents the calculation of the basic reproduction number $R_{0}$.

#### 2.2.4. Stability of the Disease-Free Equilibrium Point

**Theorem** **1.**  *The disease-free equilibrium point (DFE) is asymptotically stable if, and only if, the basic reproduction number* $R_{0}<1$.

**Proof** **of** **Theorem** **1.**  The condition of eigenvalues determines the stability of the equilibrium points. To obtain the eigenvalues, we used the Jacobian matrix and evaluated $E_{dfe}$ as follows:

(18) $$J\left(E_{dfe}\right)=\left[\begin{array}{ccccc} -B & 0 & -\beta S_{1}^{*} & 0 & 0\\ \rho & -\mu & -\left(1-e\right)\beta V_{1}^{*} & 0 & 0\\ 0 & 0 & \beta S_{1}^{*}+\left(1-e\right)\beta V_{1}^{*}-A & 0 & 0\\ 0 & 0 & \delta & -C & 0\\ 0 & 0 & \gamma_{1} & \gamma_{2} & -\mu \end{array}\right].$$

The eigenvalues are the solution of the equation

(19) $$\det\left(J-\lambda I\right)=\left(-B-\lambda \right)\left(-C-\lambda \right){\left(-\mu -\lambda \right)}^{2}\left(\beta S_{1}^{*}+\left(1-e\right)\beta V_{1}^{*}-A-\lambda \right)=0$$

The eigenvalues are $\lambda_{1}=-B<0$, $\lambda_{2}=-C<0$, $\lambda_{3,4}=-\mu <0$ and $\lambda_{5}=\beta S_{1}^{*}+\left(1-e\right)\beta V_{1}^{*}-A$. The condition that makes $\lambda_{5}=\beta S_{1}^{*}+\left(1-e\right)\beta V_{1}^{*}-A<0$ is

(20) $$\frac{\beta S_{1}^{*}+\left(1-e\right)\beta V_{1}^{*}}{A}=\frac{\beta \Lambda \left[\mu +\rho -me\mu -e\rho \right]}{\left(\delta +\varepsilon_{1}+\gamma_{1}+\mu \right)\left(\rho +\mu \right)\mu }=R_{0}<1.$$

Each eigenvalue of $J\left(E_{dfe}\right)$ has a negative real part for $R_{0}<1;$ therefore, the disease-free equilibrium point (DFE) is asymptotically stable if, and only if, $R_{0}<1$. □

#### 2.2.5. Stability of the Endemic Equilibrium Point

**Theorem** **2.**  *The endemic equilibrium point (EE) is asymptotically stable if, and only if,* $R_{0}>1$.

**Proof** **of** **Theorem** **2.**  The condition of eigenvalues determines the stability of the equilibrium points. To obtain the eigenvalues, we used the Jacobian matrix considered at $E_{end}$ as follows:

(21) $$J\left(E_{end}\right)=\left[\begin{array}{ccccc} -\beta I_{2}^{*}-B & 0 & -\beta S_{2}^{*} & 0 & 0\\ \rho & -\left(1-e\right)\beta I_{2}^{*}-\mu & -\left(1-e\right)\beta V_{2}^{*} & 0 & 0\\ \beta I_{2}^{*} & \left(1-e\right)\beta I_{2}^{*} & \beta S_{2}^{*}+\left(1-e\right)\beta V_{2}^{*}-A & 0 & 0\\ 0 & 0 & \delta & -C & 0\\ 0 & 0 & \gamma_{1} & \gamma_{2} & -\mu \end{array}\right].$$

The eigenvalues are the solution of the equation

(22) $$\det\left(J-\lambda I\right)=\left(-C-\lambda \right)\left(-\mu -\lambda \right)\left(-\lambda^{3}-p\lambda^{2}-q\lambda -r\right)=0$$

where $p=\left(2-e\right)\beta I_{2}^{*}+B+\mu$,

$q=\left(1-e\right)\beta^{2}I_{2}^{*}{}^{2}+\mu \beta I_{2}^{*}+\left(1-e\right)B\beta I_{2}^{*}+\mu B+\beta^{2}I_{2}^{*}S_{2}^{*}+{\left(1-e\right)}^{2}\beta^{2}I_{2}^{*}V_{2}^{*}$,
and $r=\left(1-e\right)\rho \beta^{2}I_{2}^{*}S_{2}^{*}+\left(1-e\right)\beta^{2}AI_{2}^{*}{}^{2}+\mu \beta^{2}I_{2}^{*}S_{2}^{*}+{\left(1-e\right)}^{2}\beta^{2}BI_{2}^{*}V_{2}^{*}$.

The eigenvalues are $\lambda_{1}=-C<0$, $\lambda_{2}=-\mu <0$ and roots of $-\lambda^{3}-p\lambda^{2}-q\lambda -r$. Through the Routh–Hurwitz criterion [28], all eigenvalues have a negative real part if p,q,r>0, and pq−r>0. Therefore, the condition that makes p>0, q>0, and r>0 is $I_{2}^{*}>0$. Then,

(23) $$-b+\sqrt{b^{2}-4ac}>0.$$

If b≤0, we obtain $-b+\sqrt{b^{2}-4ac}>0$. If b>0, we derive that $\sqrt{b^{2}-4ac}>b>0$ and then ac<0. Since a>0, we obtain that

(24) $$c=\frac{AB\mu }{\beta }-\left(1-m\right)\Lambda \mu -\left(1-e\right)\Lambda \rho -\left(1-e\right)\Lambda m\mu <0,$$

which can be derived as

(25) $$\frac{AB\mu }{\beta }-\Lambda \left[\mu +\rho -me\mu -e\rho \right]<0.$$

Then, we obtain

(26) $$R_{0}=\frac{\beta \Lambda \left[\mu +\rho -me\mu -e\rho \right]}{\left(\delta +\varepsilon_{1}+\gamma_{1}+\mu \right)\left(\rho +\mu \right)\mu }>1.$$

Considering pq−r, we obtain $pq-r=\left(2-e\right)\left(1-e\right)\beta^{3}I_{2}^{*}{}^{3}+\left(3-2e\right)\mu \beta^{2}I_{2}^{*}{}^{2}\;+\beta^{3}I_{2}^{*}{}^{2}S_{2}^{*}+\left(3-e\right)\left(1-e\right)\beta^{2}BI_{2}^{*}+\left(4-2e\right)\mu \beta BI_{2}^{*}+\left(2-e\right){\left(1-e\right)}^{2}\beta^{3}I_{2}^{*}{}^{2}V_{2}^{*}+\mu^{2}\beta I_{2}^{*}\;+2\mu^{2}B+{\left(1-e\right)}^{2}\mu B^{2}I_{2}^{*}V_{2}^{*}+\left(1-e\right)\beta B^{2}I_{2}^{*}+\mu B^{2}+\rho e\beta^{2}I_{2}^{*}S_{2}^{*}$ which is positive when $I_{2}^{*}>0,$ the same condition as for p,q,r>0. Each eigenvalue of $J\left(E_{end}\right)$ has a negative real part for $R_{0}>1$; therefore, the endemic equilibrium point (EE) is asymptotically stable if, and only if, $R_{0}>1$. □

#### 2.2.6. Sensitivity Analysis

To better test the sensitivity of the parameters of the model, a sensitivity analysis was performed to test the impact of the input parameters related to the basic reproduction number $\left(R_{0}\right),$ which determines the endemic behavior [29]. The increase in $R_{0}$ indicates the start of the pandemic. The basic reproduction number of the *SVIHR* model as shown in Equation (17) can be refined as follows:

(27) $$R_{0}=\frac{\beta \Lambda \left[\mu +\rho -me\mu -e\rho \right]}{\left(\delta +\varepsilon_{1}+\gamma_{1}+\mu \right)\left(\rho +\mu \right)\mu }=\frac{\beta \Lambda \left[\mu \left(1-me\right)+\rho \left(1-e\right)\right]}{\left(\delta +\varepsilon_{1}+\gamma_{1}+\mu \right)\left(\rho +\mu \right)\mu }.$$

Since $R_{0}$ depends on nine different positive parameters, the sensitivity indices of $R_{0}$ for each parameter are calculated based on the normalized forward sensitivity index as described in [29]. The calculation of this index is the ratio of the relative change in $R_{0}$ to the relative change in each parameter, as follows:

(28) $$\Upsilon_{\Lambda }^{R_{0}}=\left(\frac{\partial R_{0}}{\partial \Lambda }\right)\left(\frac{\Lambda }{R_{0}}\right)=1>0,$$

(29) $$\Upsilon_{\beta }^{R_{0}}=\left(\frac{\partial R_{0}}{\partial \beta }\right)\left(\frac{\beta }{R_{0}}\right)=1>0,$$

(30) $$\Upsilon_{m}^{R_{0}}=\left(\frac{\partial R_{0}}{\partial m}\right)\left(\frac{m}{R_{0}}\right)=-\frac{me\mu }{\left[\mu \left(1-me\right)+\rho \left(1-e\right)\right]}<0$$

(31) $$\Upsilon_{e}^{R_{0}}=\left(\frac{\partial R_{0}}{\partial e}\right)\left(\frac{e}{R_{0}}\right)=-\frac{e\left[m\mu +\rho \right]}{\left[\mu \left(1-me\right)+\rho \left(1-e\right)\right]}<0$$

(32) $$\Upsilon_{\delta }^{R_{0}}=\left(\frac{\partial R_{0}}{\partial \delta }\right)\left(\frac{\delta }{R_{0}}\right)=-\frac{\delta }{\left(\delta +\varepsilon_{1}+\gamma_{1}+\mu \right)}<0$$

(33) $$\Upsilon_{\varepsilon_{1}}^{R_{0}}=\left(\frac{\partial R_{0}}{\partial \varepsilon_{1}}\right)\left(\frac{\varepsilon_{1}}{R_{0}}\right)=-\frac{\varepsilon_{1}}{\left(\delta +\varepsilon_{1}+\gamma_{1}+\mu \right)}<0$$

(34) $$\Upsilon_{\gamma_{1}}^{R_{0}}=\left(\frac{\partial R_{0}}{\partial \gamma_{1}}\right)\left(\frac{\gamma_{1}}{R_{0}}\right)=-\frac{\gamma_{1}}{\left(\delta +\varepsilon_{1}+\gamma_{1}+\mu \right)}<0$$

(35) $$\Upsilon_{\mu }^{R_{0}}=\left(\frac{\partial R_{0}}{\partial \mu }\right)\left(\frac{\mu }{R_{0}}\right)=-\frac{\rho \left(1-e\right)}{\mu \left(1-me\right)+\rho \left(1-e\right)}-\frac{\mu }{\delta +\varepsilon_{1}+\gamma_{1}+\mu }-\frac{\mu }{\rho +\mu }<0$$

(36) $$\Upsilon_{\rho }^{R_{0}}=\left(\frac{\partial R_{0}}{\partial \rho }\right)\left(\frac{\rho }{R_{0}}\right)=-\frac{\rho e\mu \left(1-m\right)}{\left[\mu \left(1-me\right)+\rho \left(1-e\right)\right]\left(\rho +\mu \right)}<0$$

The parameters with a positive value of the sensitivity indices, Λ and β, have a positive effect on the basic reproduction number. This means that the increases in Λ and β can lead to an outbreak. In contrast, the increase in parameters with negative sensitivity indices values may reduce the pandemic. Therefore, increasing one of these parameters, m, ρ, e, β, $\gamma_{1},\gamma_{2}$, $\varepsilon_{1},\varepsilon_{2}$, δ, and μ, will result in reducing the pandemic. Further calculations and analyses with the numerical values of each parameter after the parameter estimations and the simulation results were performed, as explained in Section 3.

### 2.3. Data Collection and Implementation

The numbers of hospitalized and vaccinated cases from the Department of Disease Control, Ministry of Public Health, Thailand [14] from 19 July 2021 (Day 1) to 1 October 2022 (Day 440) were used to fit the unknown parameters to represent the vaccination policy in Thailand (Table S1). We used the natural death rate (*µ*) from the Thailand Death Rate 2015–2020, Department of Economic and Social Affairs, United Nations [30]. The probability rate of detection $\left(\delta \right)$ was retrieved from the SIDARTHE-V model [19]. The proportion of vaccinated individuals was obtained from the Our World in Data resource [31]. There are three types of vaccines explored in this study: RNA-based, non-replicating viral vector, and inactivated virus. The vaccine efficacy $\left(e\right)$ of each type was determined from [8]. The parametrization for unknown parameters was performed by minimizing the root mean square error (RMSE) between the fitted curve and the actual data for various values of the unknown parameters on the grid space. All models and simulations were performed and coded in MATLAB (Natick, MA, USA).

## 3. Results

### 3.1. Parameter Estimation and the Relationship between Ro, Transmission Rate, and Vaccine Efficacy

To parameterize the parameters related to the infected individuals $\left(I\right)$, for which there is no report, we estimated $\gamma_{1}$ and $\varepsilon_{1}$ using the data of the number of actual cases reported and fixing some parameters, which can be approximated from some studies, as mentioned in Section 2.3. The vaccine efficacy $\left(e\right)$ of each vaccine type is reported according to [8], as summarized in Table 1, and is used to determine the average efficacy of the vaccination policy that can control the spread.

The average efficacy of these three vaccines in Table 1 was calculated and used to estimate the unknown parameters. On average, for the whole population when using these three types of vaccines, the efficacy of the protection can be estimated at 83.24%, which is a similar level to that for a vaccine of an inactivated virus. The values of all parameters in the *SVIHR* model are shown in Table 2.

In Table 2, the assumed number of people flowing in the system per day with parameter Λ was based on the average number of hospitalized people per day and the proportion of vaccinated people (*m*). With the use of the parameters in Table 2, the relationship between the transmission rate (β), vaccine efficacy (e), and $R_{0}$ is as follows.

(37) $$R_{0}=\left(1.233344-0.7944136e\right)\beta \times 10^{6}$$

where β and e are greater than or equal to zero. If there is no vaccination policy, assuming that the vaccine efficacy (*e*) is zero, then $R_{0}=\left(1.233344\times 10^{6}\right)\beta$. Therefore, $R_{0}<1$ when $\beta <8.108038\times 10^{-7}$ and $R_{0}>1$ when $\beta >8.108038\times 10^{-7}$. Notably, this is a small number, which means that, in the case of no vaccination, a transmission rate of more than about 8 people infected per ten million (that is 0.8, or about 1 person infected per million) would cause the spread of the disease ($R_{0}>1$). If the vaccine efficacy fully terminates the disease (*e* = 1), then $R_{0}=\left(4.389308\times 10^{5}\right)\beta$. Therefore, $R_{0}<1$ when $\beta <2.278263\times 10^{-6}$ and $R_{0}>1$ when $\beta >2.278263\times 10^{-6}$. This means that, in the case of 100% efficacy of the vaccine, a transmission rate of less than about three people infected per million can converge to a disease-free equilibrium ($R_{0}<1$).

**Figure 2:**
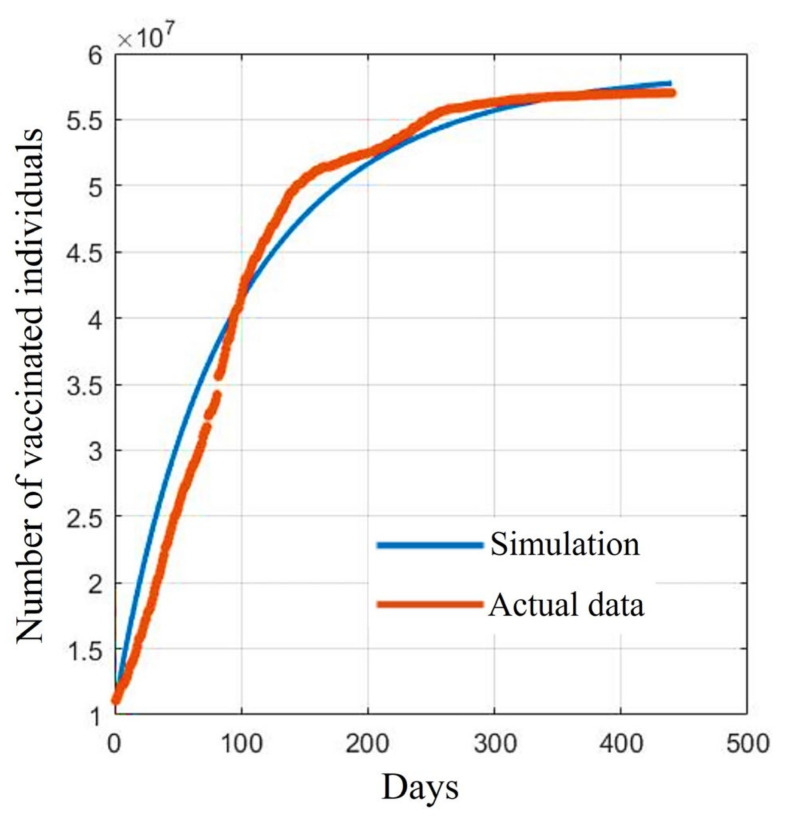
Comparison between the reported data and the fitted data of vaccinated individuals.

The fitted curves of vaccinated individuals, where *e* is the average value from Table 1 and the other parameters are from Table 2, are shown in Figure 2. This suggests that the developed model could be a suitable tool for explaining the vaccination situation in Thailand.

Figure 2 shows a comparison between the reported data and the fitted curves of the *V* population in the *SVIHR* model using the average of the vaccine efficacy and the case reports in Thailand. As in Thailand, we tried to assess possible varieties of available vaccines at that time to control the spread of the disease. We could not measure the exact proportions of the population receiving different vaccine types. To estimate the parameters, we then took the average efficacy to search for suitable values of the unknown parameters in the *SVIHR* model.

### 3.2. Simulation and Equilibrium Points

#### 3.2.1. Simulations of the *SVIHR* Model with Different Types of Vaccine

**Figure 3:**
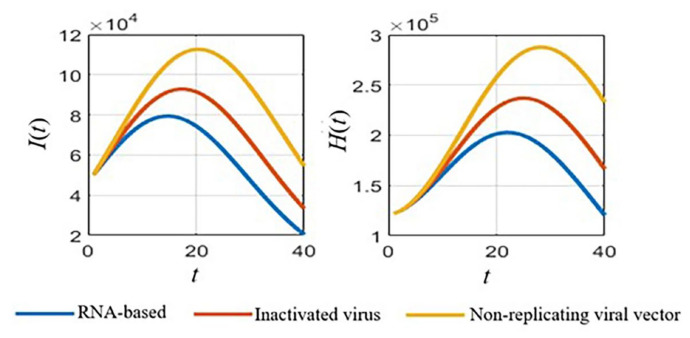
Simulated results for different vaccine types of the *SVIHR* model.

With defined parameters (Section 3.1 Table 2), we simulated the numerical results of the *SVIHR* model for different vaccine types with the fixed vaccination rate, as shown in Figure 3. An RNA-based vaccine with the highest efficacy proved the best in reducing the number of infected people and the vaccine with a non-replicating viral vector had the lowest efficacy in reducing the number of infected people. However, with the limitations of the RNA-based vaccine at that time, various vaccine types should be applied. The analysis of different vaccine efficacies and the vaccination rate is a useful tool to assess the tendency of the spread and to plan control policies, which can be simulated with the *SVIHR* model. The equilibrium points of the populations in the model for different policies (with differences in vaccine efficacies and vaccination rates) can provide suitable parameters for the model. This can be applied to the real situation enabling design of a specific plan for controlling the spread of the disease. The subsequent sections demonstrate the simulation of different scenarios which could be obtained from the *SVIHR* model.

#### 3.2.2. Simulation of the *SVIHR* Model with Various Transmission Rates and Vaccine Efficacy

To simulate the population growth compared with the equilibrium points depending on the value of $R_{0}$ and the efficacy of the vaccine $\left(e\right)$. Let us assume the initial population is as follows:

$$S\left(0\right)=80,000,\;V\left(0\right)=5000,\;I\left(0\right)=20,\;H\left(0\right)=3000\;\mathrm{and}\;R\left(0\right)=0.$$

**Figure 4:**
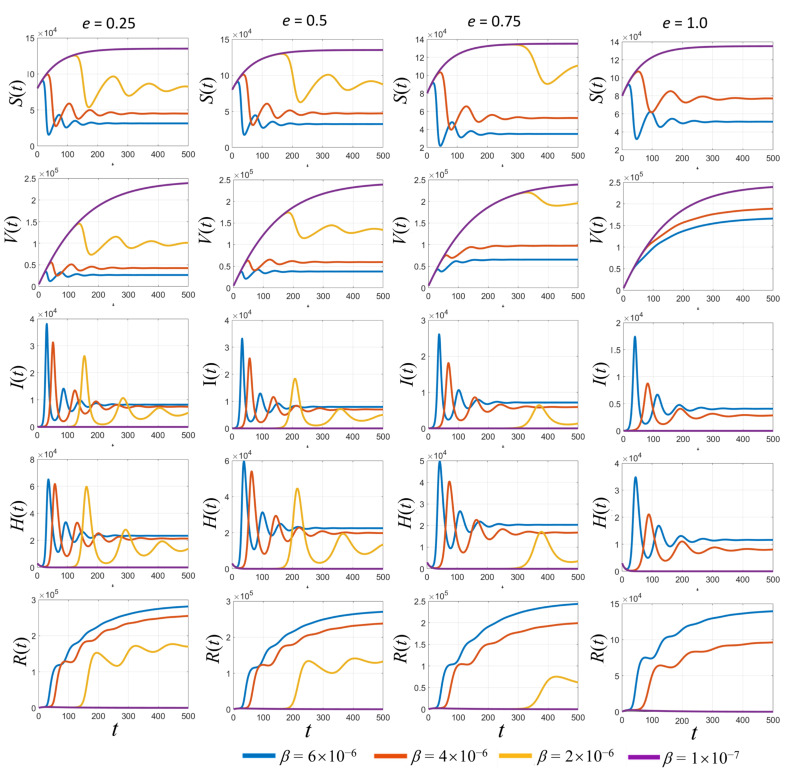
Simulated results of each population with different values of vaccine efficacy (*e*) and various transmission rates (*β*).

A broad overview of the dynamic simulations for various values of transmission rates (β) and the vaccine efficacy (*e*) is shown in Figure 4. In Figure 4, the simulated results based on the estimated parameters in Table 2 are displayed. Each row represents each subpopulation, and each column represents graphical curves of each vaccine efficacy (*e*) where each curve represents the transmission rate (β). With a low transmission rate at 2 × 10^−6^ (yellow curves in Figure 4), the results show some peaks in infected populations. When the vaccine efficacy is higher, the peaks of the infected populations are lower. Clearly, the higher the vaccine efficacy, the faster the growth of the vaccinated people curves. Finally, it also reduces the number of recovered people and hospitalized people due to the lower number of infected people over the long-term.

### 3.3. Sensitivity of Parameters and Impact on the Pandemic

By applying the parameters used in Table 2 and the sensitivity analysis in Section 2.2.6, the exact sensitivity indices values for each parameter can be calculated, as shown in Table 3. To analyze the impact of the input parameters related to the basic reproduction number $\left(R_{0}\right)$, as explained in Section 2.2.3, Λ and β have a positive effect on the basic reproduction number, while m, ρ, e, β, $\gamma_{1},\gamma_{2}$, $\varepsilon_{1},\varepsilon_{2}$, δ, and μ result in reducing the effect of the pandemic. The calculation results of the sensitivity indices in Table 3 show the exact values with the estimated parameters provided in Table 2.

**Figure 5:**
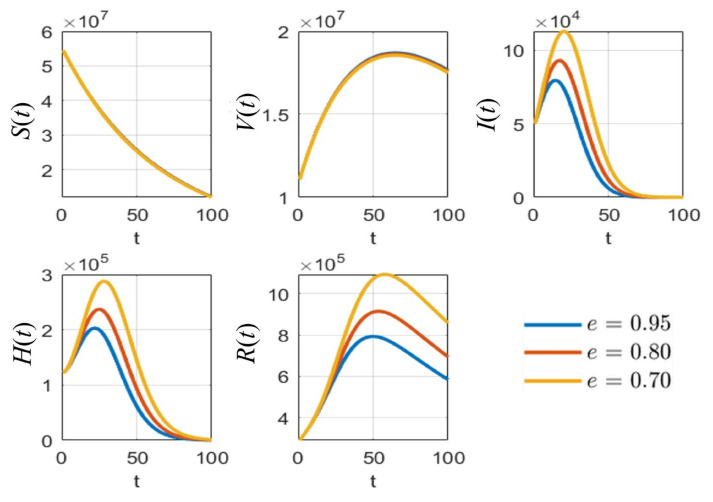
The simulation results of all populations compared with different values of vaccine efficacy $\left(e\right)$.

**Figure 6:**
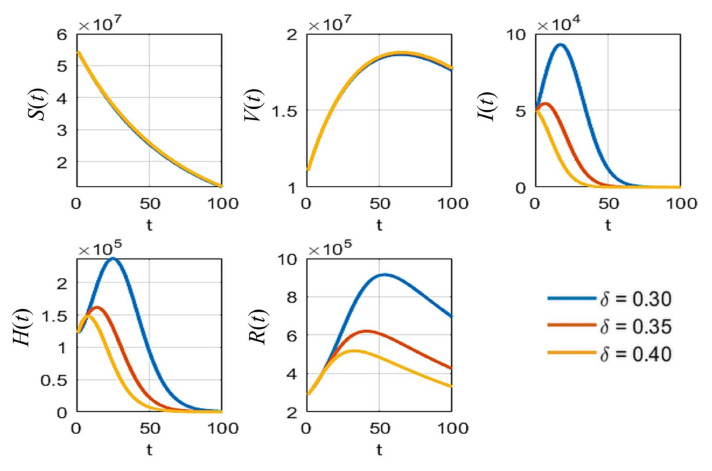
The simulation results of all populations compared with different rates of detection $\left(\delta \right)$.

**Figure 7:**
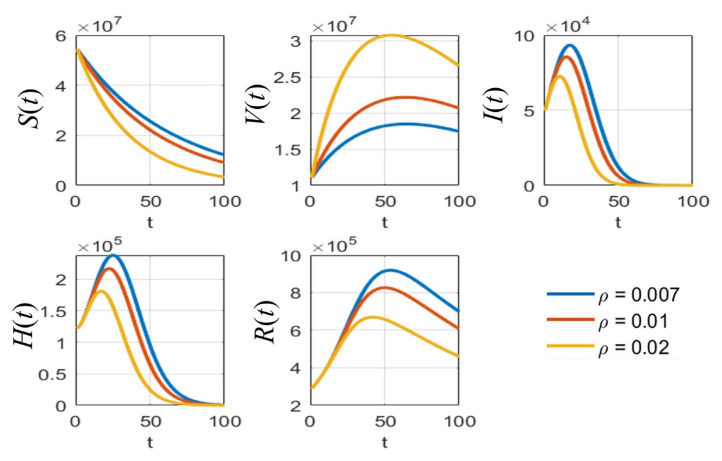
The simulation results of all populations compared with different vaccination rates $\left(\rho \right)$.

According to Table 3, the sensitivity indices values are sorted in descending order: the vaccine efficacy $\left(e\right)$ is the most effective for reducing the pandemic as it has the highest absolute sensitivity index. The second highest is the natural death rate $\left(\mu \right)$, which is hard to define and to determine in reality. It can be omitted because no application could be controlled in the same manner in the real situation. The probability rate of detection $\left(\delta \right)$ is the next parameter of interest because it can be increased by increasing the number of tests performed over the population. Next, the rate of vaccination $\left(\rho \right)$ is an obvious parameter that can be managed in practice when simulating the results of various values of this parameter. Thus, the parameters e, δ, and ρ were analyzed with various values to compare the effect on each population, as shown in Figure 5, Figure 6 and Figure 7, respectively.

As shown in Figure 5, it is clear that, with different values of vaccine efficacies, the curves for vaccinated people grow in the same manner (*V*(*t*) in Figure 5). Higher efficacies are the best for reducing the number of infected people. Figure 6 depicts the growth curves of each subpopulation according to the different rates of detection $\left(\delta \right)$. Similar to the vaccine efficacy, higher detection rates are better at reducing the number of infected people. The increase in vaccinated people directly depends on the vaccination rate $\left(\rho \right)$, as shown by the *V*(*t*) curves in Figure 7. Higher vaccination rates are better at reducing the number of infected people.

### 3.4. Trade-Off between Vaccine Efficacy and Vaccination Rate

**Figure 8:**
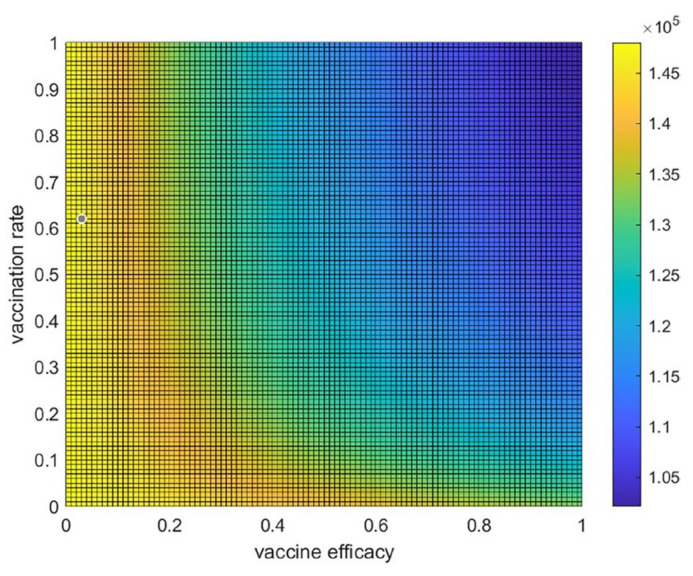
The trade-off between vaccine efficacy and the vaccination rate. Heatmap or two-dimensional plot of the average number of hospitalized individuals per day from the trade-off between vaccine efficacy and vaccination rate.

To study the trade-off between vaccine efficacy and vaccination rate, the average number of hospitalized individuals per day for each combination of the various values of the vaccination rate $\left(\rho \right)$ and the vaccine efficacy $\left(e\right)$ can be displayed as a heatmap plot, as shown in Figure 8. These values of average hospitalized individuals per day are shown by the color bar. This suggests that, when both the vaccine efficacy and the vaccination rate are greater than 0.3, the number of hospitalized people could generally be reduced. If there is high vaccine efficacy, a vaccination rate of approximately 0.15 would be beneficial for reducing the number of infected people or hospitalized people. In contrast, with a high vaccination rate (more than 0.7), the vaccine efficacy should be more than 0.4 to reduce the number of infected people. As shown in the dark blue area in Figure 8, to best control the spread, a high vaccine efficacy of about 80%, with a vaccination rate of more than 0.4, is suggested to reduce the number of infected people. If an optimal point for reducing the number of hospitalized people by more than 25% is set from starting cases of about 1.5 × 10^5^ cases to 1.1 × 10^5^ cases (1.5/1.1 ≈ 0.26), then a high vaccination rate of more than 80% would still need up to 65% vaccine efficacy to reduce the number of hospital cases by about 25%.

## 4. Discussion

The developed model, named the susceptible-vaccinated-infected-hospitalized-recovered (*SVIHR*) model, contains only six subpopulations to study the efficacy of vaccines in controlling the spread of COVID-19 in Thailand. The advantage of this simple model is that it provides valuable insights into how to apply different types of vaccines and the rate of vaccination. We tried to make the model as simple as possible to reduce the number of free parameters. The estimated parameters can be used to reflect the current situation that the government needs to manage and develop a policy to protect citizens from the disease, especially in Thailand, where there are mixed types of vaccines available for the population. Imitating the effect of low vaccine efficacy, the model demonstrates that it is quite hard to control the disease and that a high vaccination rate is urgently needed at that time. As a result, if low vaccine efficacy occurred together with low risk, then large numbers of people could then be vaccinated. However, due to the lack of some specific data related to the spread, there are still some components, such as the probability of reinfection of recovered individuals and the latent period during the spread of the disease, which should be included in the model to make it more realistic. This *SVIHR* model demonstrated non-negativity of all populations, after analysis in terms of equilibrium points and basic reproductive numbers, as well as parameter sensitivity analysis.

Furthermore, the estimation of unknown parameters in the model was then calculated by fitting a model to the reported data of vaccinated people using the average vaccine efficacy and case reports in Thailand. The number of vaccinated people is the most reliable piece of information, which can be recorded because the data can be exactly counted and collected, without being affected by other influencing factors, to reflect the number of vaccinated people. The numbers of hospitalized cases and recovered cases were also collected at the same time. However, these two numbers could be sensitive to change with various factors, such as the number of people or patients who would like to see a doctor and want to test for COVID-19, the number of field hospitals, government policy, etc.

Several qualitative mathematical models that consider the availability of vaccinations for susceptible individuals have recently been proposed. Among these models, Diagne et al. [25] investigated a mathematical model for COVID-19 that includes vaccination and treatment. The model divides the population into seven epidemiological states based on individuals’ health status: susceptible, vaccinated, exposed, symptomatic infected, asymptomatic infected, hospitalized, and recovered individuals. By fitting the model to the early COVID-19 epidemic in Senegal and running numerical simulations to optimize control using a set of parameter values, the study found that COVID-19 could be controlled in the community through the implementation of vaccination and treatment. Similar to our model, disease control can be achieved when the reproduction number is below one for the disease-free equilibrium. Additionally, the study revealed that, if the vaccine efficacy is low causing the disease reproduction number to be high, the disease may persist, even if a large proportion of the population is vaccinated. Giulia et al. [19] developed a vaccination model for the COVID-19 pandemic, namely the SIDARTHE-V model, using case reports in Italy. They expanded the SIDARTHE2 model [32] by including the effects of vaccination. The authors assumed that no reinfections would occur and that the vaccine would be fully effective against SARS-CoV-2 variants, providing full protection against death and hospitalization three weeks after vaccination. The study found that vaccination resulted in a net reduction in deaths and hospitalizations compared to scenarios without vaccination. However, the difference in effect between slow and fast vaccination was moderate. Therefore, the speed of vaccination became more important with a higher reproductive number, potentially resulting in many more deaths. Further, in 2022, studies by Arshad et al. [33] in Tunisia and Algarni et al. [34] in Saudi Arabia demonstrated the benefits of a comprehensive vaccination campaign in reducing deaths and infections associated with COVID-19 [33] and identified the vaccination rate as a crucial factor in controlling virus spread [34]. Many studies have yet to establish criteria for assessing the effectiveness of different types of vaccines. Nonetheless, given the emergence of SARS-CoV-2 variants, investigating vaccine efficacy has become a priority for present and future vaccination policies.

M.A. Acuna-Zegarra et al. [35] proposed a compartmental model to study optimal COVID-19 vaccination policies during the pandemic and vaccination period worldwide, based on natural and vaccine-induced immunity responses. Their study found that vaccine efficacy has a significant impact on optimal policy design. Higher vaccine efficacy leads to earlier implementation of the vaccination program. The study also revealed that, in the absence of vaccination, hospital occupancy can exceed 90% of beds, while the controlled dynamics considered in the study resulted in hospital occupancy decreasing to around 63% of beds. Additionally, their study identified vaccine efficacy as a key factor in determining the start of a massive vaccination strategy, while the natural immunity period determines the latter. Based on their simulations, the most significant factors in mitigating COVID-19 are the response of vaccine-induced immunity and the duration of reinfection periods [35]. Our model also reported that vaccine efficacy is an important tool to control the disease, and, if it is possible, a high vaccination rate would also help to overcome the pandemic earlier. Aruffo et al. [36] developed a model to simulate the effects of lifting nonpharmaceutical interventions under various vaccine rollout scenarios using real data from Toronto, Canada. Their findings suggest that focusing vaccination efforts on the working-age population can help curb the spread of COVID-19. Additionally, they cautioned against relaxing protective measures too soon, as new variants, vaccine efficacy, and the duration of full immunity affect the success of vaccination strategies. In addition to vaccine efficacy, vaccination policy must also account for factors such as vaccination program design and optimal dosing strategies. Recent research has underscored the value of modeling and simulation studies for exploring the potential impact of double doses [37,38].

The studies of Paul et al. [37] and Peter et al. [38] have also shown that a full vaccination program can significantly reduce cases and impact disease eradication. Public health professionals can use this information to effectively control COVID-19 outbreaks in Bangladesh [37] and Malaysia [38]. Overall, mass vaccination exercises are crucial in achieving herd immunity and preventing further outbreaks of COVID-19. Designing a successful vaccination policy requires balancing the trade-off between vaccination rate and vaccine efficacy. While the vaccination rate is crucial, it depends on the efficacy of the vaccine and the number of doses required. Achieving full vaccination of the population and 100% vaccine efficacy in the face of a pandemic is impractical. As a result, decision-makers must consider this trade-off to make informed choices about vaccination policies.

In our study, the trade-off between the vaccine efficacy and the vaccination rate has been examined, which explicitly demonstrated that, even with quite low vaccine efficacy, it is still possible to control the disease with a moderate rate of vaccination in Thailand. The heatmap analysis would exhibit the same tendency but different results for a suitable combination of vaccination rate and vaccine efficacy to control the disease in different countries which have different circumstances. It is clear that high rates of vaccination and high vaccine efficacy are the best approaches to control disease, which is quite hard in practice when a new pandemic occurs and there is no effective vaccine available. The recognition of this trade-off could help to determine a suitable rate of vaccination required in a certain situation. Thus, the study of vaccination rate and vaccine efficacy is still of interest for designing a control policy. Various types of vaccines for different SARS-CoV2 variants have different efficacies and involve different levels of expenditure that affect policy. It will provide a very valuable tool in the end; the development of a mathematical model to cope with this specific task should be accomplished soon.

## 5. Conclusions

In sum, in this study, we formulated a mathematical model of the COVID-19 transmission mechanism for the control of vaccination for susceptible individuals in Thailand, namely, the *SVIHR* model. This model consists of susceptible individuals $\left(S\right)$, vaccinated individuals $\left(V\right)$, infected individuals $\left(I\right)$, hospitalized individuals $\left(H\right)$, and recovered individuals $\left(R\right)$. We theoretically analyzed the nature of the model parameters and demonstrated that the number of each population was non-negative. Two equilibrium points were obtained: the disease-free equilibrium point (DFE) and the endemic equilibrium point (EE). The basic reproduction number $\left(R_{0}\right)$ was obtained using a next-generation matrix to determine the stability of the equilibriums. Both the disease-free and endemic equilibrium are globally asymptotically stable when the effective reproduction number $R_{0}$ is less than or greater than unity. The derived critical vaccination threshold is dependent on the vaccine efficacy for disease eradication whenever $R_{0}>1$, even if vaccine coverage is high. By applying the actual data from the Department of Disease Control, Ministry of Public Health, Thailand, the fitted parameters were provided and used in the actual analysis. A sensitivity analysis gave the most relevant parameters to reduce the pandemic, which were the vaccine efficacy $\left(e\right)$, the probability rate of detection $\left(\delta \right)$, and the vaccination rate $\left(\rho \right)$. With the trade-off analysis, a high vaccination rate still required up to 65% efficacy to reduce the number of hospital cases by about 25%. Therefore, the comparison between the vaccine efficacy and the vaccination rate proved that vaccine efficacy is the essential factor to control the spread of COVID-19 in Thailand. The particular value of this model is as a useful tool for designing and creating vaccination policies in Thailand. The implementation of optimal points between vaccine efficacy and vaccination rate for a certain period could be carried out to reduce the number of hospitalizations. Based on the use of different vaccine types in Thailand at the time of the study, the model can be adapted to plan for various proportions of each vaccine type to help in designing policy in advance. The approach in this study can be used as a simple model for a vaccination hub with different vaccine types and vaccination rates to determine control policy. Moreover, it can be developed further in future studies to include both therapeutic and nontherapeutic measures of the dynamics of COVID-19, as well as other diseases.

## Figures and Tables

**Table 1:** The vaccine efficacy depends on the type of vaccine.

Vaccine Types	Vaccine Efficacy (*e*)	Reference
RNA-based	0.9572	[8]
Non-replicating viral vector	0.7103	[8]
Inactivated virus	0.8350	[8]
Average	0.8324	

**Table 2:** The value of each parameter in the *SVIHR* model for the dynamic simulation to investigate the long-term behavior.

Parameter	Value	Reference
Λ	3000 individuals/day	Assumed
m	0.3235	[31]
ρ	0.007117	[14]
$\gamma_{1}$	$7.4352\times 10^{-8}$	Estimated
$\gamma_{2}$	0.09673	[14]
$\varepsilon_{1}$	$2.887\times 10^{-15}$	Estimated
$\varepsilon_{2}$	0.001069	[14]
δ	0.3	[19]
μ	0.0079	[30]

**Table 3:** Sensitivity indices of $R_{0}$ for each parameter using the estimated values detailed in Section 3.2.

Parameters	Sensitivity Index
Λ	1
β	1
$\varepsilon_{1}$	$-3.7782\times 10^{-8}$
$\gamma_{1}$	$-2.0857\times 10^{-7}$
ρ	−0.30410
m	−0.30684
δ	−0.97434
μ	−0.99814
e	−1.1613

## Data Availability

The data is provided in Supplementary Materials, Table S1.

## Supplementary Materials

The following supporting information can be downloaded at: https://www.mdpi.com/article/10.3390/tropicalmed8030175/s1, Table S1: The number of hospitalized and vaccinated cases.

## References

[1] Zhou P., Yang X.-L., Wang X.-G., Hu B., Zhang L., Zhang W., Si H.-R., Zhu Y., Li B., Huang C.-L. (2020). A pneumonia outbreak associated with a new coronavirus of probable bat origin. Nature.

[2] Huang C., Wang Y., Li X., Ren L., Zhao J., Hu Y., Zhang L., Fan G., Xu J., Gu X. (2020). Clinical features of patients infected with 2019 novel coronavirus in Wuhan, China. Lancet.

[3] World Health Organization WHO Coronavirus (COVID-19) Dashboard. https://covid19.who.int/.

[4] Cattaneo C., Pagliarini E., Mambrini S.P., Tortorici E., Mene R., Torlasco C., Perger E., Parati G., Bertoli S. (2022). Changes in smell and taste perception related to COVID-19 infection: A case-control study. Sci. Rep..

[5] Hu B., Guo H., Zhou P., Shi Z.L. (2021). Characteristics of SARS-CoV-2 and COVID-19. Nat. Rev. Microbiol..

[6] Van Doremalen N., Bushmaker T., Morris D.H., Holbrook M.G., Gamble A., Williamson B.N., Tamin A., Harcourt J.L., Thornburg N.J., Gerber S.I. (2020). Aerosol and Surface Stability of SARS-CoV-2 as Compared with SARS-CoV-1. N. Engl. J. Med..

[7] Dashtbali M., Mirzaie M. (2021). A compartmental model that predicts the effect of social distancing and vaccination on controlling COVID-19. Sci. Rep..

[8] Fiolet T., Kherabi Y., MacDonald C.-J., Ghosn J., Peiffer-Smadja N. (2022). Comparing COVID-19 vaccines for their characteristics, efficacy and effectiveness against SARS-CoV-2 and variants of concern: A narrative review. Clin. Microbiol. Infect..

[9] Costa Clemens S.A., Weckx L., Clemens R., Almeida Mendes A.V., Ramos Souza A., Silveira M.B.V., da Guarda S.N.F., de Nobrega M.M., de Moraes Pinto M.I., Gonzalez I.G.S. (2022). Heterologous versus homologous COVID-19 booster vaccination in previous recipients of two doses of CoronaVac COVID-19 vaccine in Brazil (RHH-001): A phase 4, non-inferiority, single blind, randomised study. Lancet.

[10] Voysey M., Clemens S.A.C., Madhi S.A., Weckx L.Y., Folegatti P.M., Aley P.K., Angus B., Baillie V.L., Barnabas S.L., Bhorat Q.E. (2021). Safety and efficacy of the ChAdOx1 nCoV-19 vaccine (AZD1222) against SARS-CoV-2: An interim analysis of four randomised controlled trials in Brazil, South Africa, and the UK. Lancet.

[11] Shay D.K., Gee J., Su J.R., Myers T.R., Marquez P., Liu R., Zhang B., Licata C., Clark T.A., Shimabukuro T.T. (2021). Safety Monitoring of the Janssen (Johnson & Johnson) COVID-19 Vaccine—United States, March-April 2021. MMWR Morb. Mortal. Wkly. Rep..

[12] Polack F.P., Thomas S.J., Kitchin N., Absalon J., Gurtman A., Lockhart S., Perez J.L., Pérez Marc G., Moreira E.D., Zerbini C. (2020). Safety and Efficacy of the BNT162b2 mRNA Covid-19 Vaccine. N. Engl. J. Med..

[13] Tran T.N.M., May B.P., Ung T.T., Nguyen M.K., Nguyen T.T.T., Dinh V.L., Doan C.C., Tran T.V., Khong H., Nguyen T.T.T. (2021). Preclinical Immune Response and Safety Evaluation of the Protein Subunit Vaccine Nanocovax for COVID-19. Front. Immunol..

[14] Ministry of Public Health COVID-19 Situation Reports. https://ddc.moph.go.th/viralpneumonia/eng/situation.php.

[15] Sadarangani M., Marchant A., Kollmann T.R. (2021). Immunological mechanisms of vaccine-induced protection against COVID-19 in humans. Nat. Rev. Immunol..

[16] Allen S.L.J. (2003). An Introduction to Stochastic Processes with Applications to Biology.

[17] Allen S.L.J. (2008). An Introduction to Stochastic Epidemic Models: Mathematical Epidemiology.

[18] Hethcote H.W. (1989). Three Basic Epidemiological Models. Applied Mathematical Ecology.

[19] Giordano G., Colaneri M., Di Filippo A., Blanchini F., Bolzern P., De Nicolao G., Sacchi P., Colaneri P., Bruno R. (2021). Modeling vaccination rollouts, SARS-CoV-2 variants and the requirement for non-pharmaceutical interventions in Italy. Nat. Med..

[20] Husniah H., Ruhanda R., Supriatna A.K., Biswas M.H.A. (2021). SEIR Mathematical Model of Convalescent Plasma Transfusion to Reduce COVID-19 Disease Transmission. Mathematics.

[21] Prathumwan D., Trachoo K., Chaiya I. (2020). Mathematical Modeling for Prediction Dynamics of the Coronavirus Disease 2019 (COVID-19) Pandemic, Quarantine Control Measures. Symmetry.

[22] Intarapanya T., Suratanee A., Pattaradilokrat S., Plaimas K. (2022). Modeling the spread of COVID-19 as a consequence of undocumented immigration toward the reduction of daily hospitalization: Case reports from Thailand. PLoS ONE.

[23] Jabal K.A., Wiegler K.B., Edelstein M. (2021). Convalescent plasma from people vaccinated after COVID-19 infection. Lancet Microbe.

[24] Nagoba B., Gavkare A., Jamadar N., Mumbre S., Selkar S. (2020). Positive aspects, negative aspects and limitations of plasma therapy with special reference to COVID-19. J. Infect. Public Health.

[25] Diagne M.L., Rwezaura H., Tchoumi S.Y., Tchuenche J.M. (2021). A Mathematical Model of COVID-19 with Vaccination and Treatment. Comput. Math. Methods Med..

[26] Leung N.H.L. (2021). Transmissibility and transmission of respiratory viruses. Nat. Rev. Microbiol..

[27] Van den Driessche P., Watmough J. (2002). Reproduction numbers and sub-threshold endemic equilibria for compartmental models of disease transmission. Math. Biosci..

[28] Bodson M. (2020). Explaining the Routh–Hurwitz Criterion: A Tutorial Presentation [Focus on Education]. IEEE Control Syst. Mag..

[29] Chitnis N., Hyman J.M., Cushing J.M. (2008). Determining important parameters in the spread of malaria through the sensitivity analysis of a mathematical model. Bull. Math. Biol..

[30] Thailand Death Rate 2015–2020. United Nations—World Population Prospects. https://population.un.org/wpp/.

[31] Hannah Ritchie E.M., Rodés-Guirao L., Appel C., Giattino C., Ortiz-Ospina E., Hasell J., Macdonald B., Beltekian D., Roser M. Coronavirus Pandemic (COVID-19). Our World Data 2020. https://ourworldindata.org/coronavirus.

[32] Higazy M. (2020). Novel fractional order SIDARTHE mathematical model of COVID-19 pandemic. Chaos Solitons Fractals.

[33] Arshad S., Khalid S., Javed S., Amin N., Nawaz F. (2022). Modeling the impact of the vaccine on the COVID-19 epidemic transmission via fractional derivative. Eur. Phys. J. Plus.

[34] Algarni A.D., Ben Hamed A., Hamdi M., Elmannai H., Meshoul S. (2022). Mathematical COVID-19 model with vaccination: A case study in Saudi Arabia. PeerJ Comput. Sci..

[35] Acuna-Zegarra M.A., Diaz-Infante S., Baca-Carrasco D., Olmos-Liceaga D. (2021). COVID-19 optimal vaccination policies: A modeling study on efficacy, natural and vaccine-induced immunity responses. Math. Biosci..

[36] Aruffo E., Yuan P., Tan Y., Gatov E., Moyles I., Belair J., Watmough J., Collier S., Arino J., Zhu H. (2022). Mathematical modelling of vaccination rollout and NPIs lifting on COVID-19 transmission with VOC: A case study in Toronto, Canada. BMC Public Health.

[37] Paul A.K., Kuddus M.A. (2022). Mathematical analysis of a COVID-19 model with double dose vaccination in Bangladesh. Results Phys..

[38] Peter O.J., Panigoro H.S., Abidemi A., Ojo M.M., Oguntolu F.A. (2023). Mathematical Model of COVID-19 Pandemic with Double Dose Vaccination. Acta Biotheor..

